# Examining the impact of 11 long-standing health conditions on health-related quality of life using the EQ-5D in a general population sample

**DOI:** 10.1007/s10198-013-0559-z

**Published:** 2014-01-10

**Authors:** Mengjun Wu, John E. Brazier, Benjamin Kearns, Clare Relton, Christine Smith, Cindy L. Cooper

**Affiliations:** 1 Institute of Mental Health, University of Nottingham, Innovation Park, Jubilee Campus, Triumph Road, Nottingham, NG7 2TU UK; 2 School of Health and Related Research, University of Sheffield, Regent Court, 30 Regent Street, Sheffield, S1 4DA UK; 3 Research and Development Department, Barnsley Hospital NHS Foundation Trust, Gawber Road, Barnsley, S75 2EP UK

**Keywords:** EQ-5D, Health-related quality of life, Chronic conditions, Health economics methods, Health surveys, UK sample, I18

## Abstract

**Objectives:**

Health-related quality of life (HRQoL) measures have been increasingly used in economic evaluations for policy guidance. We investigate the impact of 11 self-reported long-standing health conditions on HRQoL using the EQ-5D in a UK sample.

**Methods:**

We used data from 13,955 patients in the South Yorkshire Cohort study collected between 2010 and 2012 containing the EQ-5D, a preference-based measure. Ordinary least squares (OLS), Tobit and two-part regression analyses were undertaken to estimate the impact of 11 long-standing health conditions on HRQoL at the individual level.

**Results:**

The results varied significantly with the regression models employed. In the OLS and Tobit models, pain had the largest negative impact on HRQoL, followed by depression, osteoarthritis and anxiety/nerves, after controlling for all other conditions and sociodemographic characteristics. The magnitude of coefficients was higher in the Tobit model than in the OLS model. In the two-part model, these four long-standing health conditions were statistically significant, but the magnitude of coefficients decreased significantly compared to that in the OLS and Tobit models and was ranked from pain followed by depression, anxiety/nerves and osteoarthritis.

**Conclusions:**

Pain, depression, osteoarthritis and anxiety/nerves are associated with the greatest losses of HRQoL in the UK population. The estimates presented in this article should be used to inform economic evaluations when assessing health care interventions, though improvements can be made in terms of diagnostic information and obtaining longitudinal data.

## Introduction

Health-related quality of life (HRQoL) measures have been increasingly employed in economic evaluations [1–4]. Both generic and disease-specific health status instruments have been developed to measure HRQoL; however, there is no gold standard. The EQ-5D instrument is a generic HRQoL measurement [5]. Using a representative sample of the UK general population, a single index value for all the hypothetical health states described by the EQ-5D is linked by the UK EQ-5D index tariff [6, 7].

HRQoL is a useful measure for health policy guidance. Health improvement and inequality reduction in health are the main health policy targets, and both the population-level health and its distribution could be used to assess the achievement of targets. HRQoL aims to capture the elements of quality of life that have a direct impact on aspects of an individual’s perceived health such as physical, psychological, social and role functioning as well as general well-being [8]. In addition, HRQoL measurement potentially captures the following two requirements. First, measures for outcomes of health care should be multidimensional to capture the change of the philosophy of health care from illness reduction to well-being improvement. Second, generic single-dimensional utility measures are required to compare the costs and benefits of different disease treatments [9].

There have been an increasing number of studies conducted to explore the relative impacts of different chronic diseases on HRQoL; however, the results remain inconclusive. Estimates for specific conditions vary greatly with differences in methodology [10]. For instance, using data (*N* = 8,028) from Finland, the relationships between 29 chronic conditions and HRQoL were assessed using Tobit and censored least absolute deviation regression models. Musculoskeletal disorders were found to be associated with the largest losses of HRQoL, followed by psychiatric conditions [11]. The 2000 Medical Expenditure Panel Survey (MEPS) was used to explore the relationship between clinical conditions and HRQoL in the US general population using ordinary least square (OLS) regression; emphysema tended to have the greatest negative impact while asthma tended to have the least [12]. A similar finding about asthma was reported when assessing the relationship between disease and HRQoL in the Swedish general population using OLS regression [13]. Furthermore, their results also showed that mental distress had the largest negative impact.

To date, there has been little attempt to explore the impact of chronic health conditions on HRQoL using the UK general population. For example, Ara and Brazier [14] used a cardiovascular disease model and cost per quality adjusted life-year (QALY) thresholds to estimate health-state utility values for multiple health conditions using the Health Survey for England (*N* = 26,679). Similar cost per QALY results in cardiovascular disease were produced by the additive and multiplicative models; however, this finding may not be generalisable to other health conditions. Therefore, further research in other health conditions and data sets is needed.

Our aim is to examine the impact of 11 long-standing health conditions on the EQ-5D score using a large UK data set of self-reported questionnaire data containing information on both the EQ-5D and a list of long-standing health conditions. We used various regression models to account for the distributional features of the EQ-5D scores.

## Methods

### Data

Data for this study were obtained from the South Yorkshire Cohort (SYC) [15], which is a postal and online patient self-completed health questionnaire of patients aged 16 to 85 years registered with 42 GP practices in South Yorkshire, UK. The SYC protocol was approved by the NHS Research Ethics Committee on 27 April 2010 (09/H1306/97). All patients registered with the recruited GP practices aged 16 to 85 years were approached to enter the survey. Each patient received an invitation letter and a health questionnaire from their GP practice by post; however, patients were also offered access to the health questionnaire online. This study was based on a single wave data set from completed questionnaires from June 2010 to June 2012 with a response rate of 17.8 %.

In order to help improve the response rate, postage stamps rather than prepaid envelopes were used so that the envelopes appeared less official but more personal [16]. The data set had 18,093 patient observations [15]. Our analysis focussed on 13,955 patient observations with non-missing data for the variables chosen for this study.

### Dependent variable

A preference-based measure, the EQ-5D, which has five dimensions—mobility, self-care, usual activities, pain/discomfort and anxiety/depression—was used as the dependent variable. There are three levels within each dimension, namely no problems, moderate problems and severe problems; thus, in total 243 health states are defined [17]. Patients classified themselves into the EQ-5D by self-completing the questions. The EQ-5D scores ranged from −0.594 to 1. The EQ-5D usually comes with a visual analogue rating scale, but it was not included in this health questionnaire.

### Independent variables

Participants were asked “Do you have any long-standing illness, health problem, condition or disability? If yes, please tick all that apply”. The list included: pain, insomnia, anxiety/nerves, depression, diabetes, breathing problems (e.g. chronic bronchitis, asthma or emphysema), high blood pressure, heart disease, osteoarthritis, stroke and cancer. We included all health conditions and the number of comorbidities to be tested in our models (“Appendix”).

The EQ-5D scores are also known to be affected by sociodemographic factors, including age, gender, ethnicity, education and socio-economic status [11–13]. The SYC data set contained socio-demographic characteristics, namely age, gender, age and gender interaction terms, ethnicity, education, socio-economic status (occupation is used as a proxy for this variable) and current employment status, which were all included in our models.

### Analyses

First, we presented descriptive statistics of the EQ-5D, long-standing health conditions and socio-demographic characteristics of the sample, and the EQ-5D scores in the light of socio-demographic characteristics and long-standing health conditions.

Second, we employed regression models to examine the impact of long-standing health conditions on the EQ-5D scores, controlling for socio-demographic characteristics. In our data set, HRQoL measure, the EQ-5D scores had a ceiling effect with about 47 % of respondents reported full health. There have been debates on the choice of appropriate methods of analysis of censored HRQoL scores [18–21]. The starting point was to use OLS regression, which assumes a linear relationship between the dependent variable (the EQ-5D scores) and the independent variables (long-standing health conditions). However, OLS models could produce estimates >1 or <−0.594, because OLS ignores the fact that the EQ-5D is bounded between −0.594 and 1. In other words, the bounded nature of the EQ-5D scores is ignored by OLS models, which could result in biased estimates [18–21].

Because of the bounded nature of the EQ-5D scores, we also employed Tobit models, which allow for the lowest and highest EQ-5D scores so that estimates are not beyond the range of EQ-5D scores (−0.594 to 1). That is, Tobit models were used to see whether they provided better predictions compared to those by OLS models. Using Tobit coefficients to estimate predicted EQ-5D scores, we fitted a linear predictor that was adjusted according to the limits applied to the model (i.e. scores cannot be <−0.594 or >1). The interpretation for Tobit regression coefficients is done in a similar manner to OLS regression coefficients, but the linear effect is not on the observed outcome; instead it is on the uncensored latent variable [18–21].

Since approximately half of the respondents reported full health, we also used a two-part model in which two different types of model are combined to estimate different parts of the distribution of the EQ-5D scores. The first part of the model uses a logistic regression to predict the likelihood of respondents reporting full health and the second part employs a truncated OLS model to predict the EQ-5D scores of respondents reporting non-full health [22].

Models were compared using a set of criteria, including overall diagnosis by root mean squared error (RMSE) in the OLS, and sigma in the Tobit and the second part of two-part models, Akaike information criterion (AIC), Bayesian information criterion (BIC), and the relative size and significance of individual parameter estimates. The measurement of accuracy can be compared between the models using RMSE and sigma. The more accurate the model is, the smaller the error and hence the smaller the RMSE. Sigma is the estimated standard error of Tobit/second part of two-part model and is comparable to RMSE in the OLS [23].

## Results

### Descriptive statistics

**Fig. 1:**
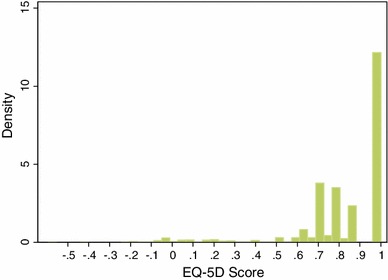
Distribution of the EQ-5D scores

Figure 1 shows the distribution of the EQ-5D scores. As illustrated, the EQ-5D scores are not normally distributed; instead the distribution is highly skewed to the right at 1. Clustering was noted in the EQ-5D scores, where there were far more scores of 1 than all other scores. There were also a relatively large number of scores ranging from 0.7 to 0.9. As shown in Table 1, the mean score [standard deviation (SD)] of EQ-5D was 0.831 (0.229), which is very close to the UK general population values of 0.853 (0.233) [24]. There were fewer respondents reporting being in full health (1.0) for the EQ-5D compared to the general population (i.e. 47 vs. 52 %). There was a similar proportion reporting their health being worse than dead compared to the general population (i.e. 2.0 vs. 1.6 %) [24].

**Table 1:** Distribution of socio-demographic characteristics, long-standing health conditions and EQ-5D scores

Variable	*N* (%)	EQ-5D score	The UK population norms
Mean EQ-5D score (SD)	13,955 (100)	0.831 (0.229)	0.853^24^
Age bands			
Younger (<41)	3,308 (23.7)	0.851 (0.222)	0.922^14^
Middle aged (41–65)	6,666 (47.8)	0.830 (0.231)	0.845^14^
Retired (66 or over)	3,981 (28.5)	0.815 (0.230)	0.741^14^
Mean age (SD)	53.9 (16.9)	0.831 (0.229)	0.843^14^
Gender			
Male	6,112 (43.8)	0.831 (0.227)	0.853^14^
Female	7,843 (56.2)	0.830 (0.230)	0.832^14^
Ethnicity			
White	13,406 (96.1)	0.831 (0.228)	
Non-white	549 (3.9)	0.822 (0.244)	
Educational attainment			
No qualifications	3,482 (25.0)	0.780 (0.266)	0.825^39^
School qualifications	10,473 (75.0)	0.848 (0.212)	0.895^39^
Degree	4,685 (33.6)	0.875 (0.190)	0.940^39^
Socio-economic status			
Blue collar	4,442 (31.8)	0.790 (0.263)	0.820^39^
White collar	9,513 (68.2)	0.850 (0.208)	0.880^39^
Current employment status			
No	6,031 (43.2)	0.779 (0.259)	
Yes	7,924 (56.8)	0.870 (0.193)	
Long-standing health conditions			
No health condition	6,026 (43.2)	0.942 (0.108)	
High blood pressure	2,532 (18.1)	0.731 (0.279)	
Cancer	386 (2.8)	0.725 (0.262)	
Breathing problems	1,394 (10.0)	0.694 (0.308)	
Diabetes	823 (5.9)	0.681 (0.302)	
Heart disease	774 (5.5)	0.656 (0.286)	
Stroke	246 (1.8)	0.610 (0.328)	
Osteoarthritis	1,222 (8.8)	0.587 (0.288)	
Insomnia	870 (6.2)	0.570 (0.331)	
Anxiety/nerves	1,265 (9.1)	0.569 (0.319)	
Pain	2,671 (19.1)	0.559 (0.290)	
Depression	1,090 (7.8)	0.526 (0.338)	
Number of comorbidities			
No	10,517 (75.4)	0.902 (0.145)	
Yes (1 or more)	3,438 (24.6)	0.612 (0.291)	

Table 1 presents respondents’ socio-demographic characteristics. The mean age (SD) was 53.9 (16.9) and 43.8 % were male. It was observed that there are more younger and middle-aged people (<41 and 41–65 years) reporting being in full health (1.0) than retired people (66 or over) (i.e. 35 vs. 12 %). This indicates that the EQ-5D scores vary by age. However, there were similar proportions of reporting health being worse than dead (<0) between younger and middle aged people and retired people (i.e. 1.5 vs. 0.6 %). Furthermore, there were no large gender differences in terms of reporting being in full health (1.0) and being worse than dead (<0) between males and females (i.e. 20.6 vs. 26.6 % and 0.9 vs. 1.1 %).

In addition, Table 1 shows the EQ-5D scores in the light of socio-demographic characteristics and long-standing health conditions. Males had similar scores as females, and whites had higher scores than non-whites. Compared to respondents with no qualifications, respondents with higher qualifications had higher scores, and the greatest difference was between respondents with a degree and those with no qualifications. White collar workers and currently employed respondents had higher scores than blue collar workers and currently unemployed ones respectively. Scores for respondents with long-standing health conditions ranged from 0.526 to 0.731, where respondents with depression had the lowest scores and respondents with high blood pressure had the highest scores. In contrast, respondents with no health conditions had higher scores (0.942). Respondents with no comorbidity had scores almost close to 1 (0.902), while respondents with one or more comorbidities had lower scores (0.612).

### Regression results

Table 2 presents the relationship between self-reported long-standing health conditions and the EQ-5D scores using OLS and Tobit regression models. RMSE is lower than sigma, which indicates that OLS models are more accurate than Tobit models. Furthermore, according to AIC and BIC figures, it indicates that in OLS and Tobit models including socio-demographics improved goodness of fit and overall OLS models provide better goodness of fit.

**Table 2:** OLS and Tobit regression models: estimating the EQ-5D scores

Explanatory variables	Model 1 OLS Health conditions without socio-demographics		Model 1 OLS Health conditions with socio-demographics		Model 2 Tobit Health conditions without socio-demographics		Model 2 Tobit Health conditions with socio-demographics	
	*β*	SE	*β*	SE	*β*	SE	*β*	SE
Pain	−0.235***	0.004	−0.229***	0.004	−0.318***	0.007	−0.308***	0.007
Insomnia	−0.061***	0.006	−0.059***	0.006	−0.066***	0.010	−0.064***	0.010
Anxiety/nerves	−0.106***	0.006	−0.105***	0.006	−0.147***	0.009	−0.144***	0.009
Depression	−0.172***	0.006	−0.173***	0.006	−0.212***	0.010	−0.215***	0.010
Diabetes	−0.055***	0.006	−0.052***	0.006	−0.072***	0.011	−0.068***	0.010
Breathing problems	−0.059***	0.005	−0.056***	0.005	−0.077***	0.008	−0.071***	0.008
High blood pressure	−0.031***	0.004	−0.024***	0.004	−0.046***	0.007	−0.034***	0.007
Heart disease	−0.056***	0.007	−0.050***	0.007	−0.077***	0.011	−0.066**	0.011
Osteoarthritis	−0.113***	0.006	−0.108***	0.006	−0.154***	0.009	−0.146***	0.009
Stroke	−0.048***	0.011	−0.043***	0.011	−0.043**	0.018	−0.035**	0.018
Cancer	−0.032***	0.009	−0.027***	0.009	−0.052***	0.015	−0.044***	0.014
Number of comorbidities	0.003	0.006	0.005	0.006	−0.029***	0.010	−0.025**	0.010
Control variables								
Age bands								
41–65			−0.005	0.004			−0.018**	0.008
66 or over			−0.005	0.005			−0.021**	0.009
Male			0.0002	0.006			−0.004	0.011
Male* 41–65			0.003	0.007			0.011	0.013
Male* 66 or over			0.0003	0.008			0.005	0.014
White			0.003	0.007			0.010	0.013
School qualifications			0.016***	0.003			0.023***	0.006
Degree			0.018***	0.003			0.043***	0.006
White collar			0.018***	0.003			0.023***	0.006
Currently employed			0.026***	0.003			0.049***	0.005
Observations	13,955		13,955		13,955		13,955	
*p* value	0.000		0.000		0.000		0.000	
*R* ^2^	0.473		0.482					
Pseudo *R* ^2^					0.434		0.452	
RMSE	0.166		0.165					
Sigma					0.263		0.260	
AIC	−10,488		−10,723		9,178		8,919	
BIC	−10,389		−10,549		9,284		9,100	

Reference categories: no health condition, no comorbidity, age band 15–40, female, non-white, no qualification, below degree, blue collar, currently unemployed

*SE* standard error

** *p* < 0.05; *** *p* < 0.01

As expected, the predicted EQ-5D scores decreased with all long-standing health conditions and for all health conditions the decrements were statistically significant in both regression models. Scores decreased most for respondents with pain, indicating that pain tended to have the largest negative impact. This was followed by depression, osteoarthritis and anxiety/nerves. In contrast, scores decreased least for respondents with high blood pressure. Inclusion of socio-demographic characteristics changed the coefficients for self-reported health conditions relatively little for both models, suggesting that the relationships of socio-demographic characteristics and self-reported health conditions with the predicted EQ-5D scores were independent with each other. Therefore, for simplicity we focussed on the models without socio-demographics.

In the OLS model, scores reduced by 0.235 for respondents with pain, and 0.172, 0.113 and 0.106 for respondents with depression, osteoarthritis and anxiety/nerves respectively. Respondents with high blood pressure led to reductions in scores by 0.031 only. Compared to the OLS model, the Tobit model produced reductions in scores by 0.318 for respondents with pain, and 0.212, 0.154 and 0.147 respectively for respondents with depression, osteoarthritis and anxiety/nerves. Scores reduced by 0.046 for respondents with high blood pressure. Overall, we observed that Tobit models generated greater decreases in scores for respondents with all long-standing health conditions. Furthermore, number of comorbidities is statistically significant only in the Tobit model. Scores reduced by 0.029 for respondents with one or more comorbidities.

In relation to socio-demographic characteristics, in the OLS model, higher educational attainment was associated with slightly higher scores. White collar workers and currently employed respondents had slightly higher scores than blue collar workers and currently unemployed ones respectively. Age, gender, age and gender interaction terms and ethnicity were, however, not statistically significant. In the Tobit model, we noticed similar findings about educational attainment, socio-economic status and current employment status, but they were associated with higher scores compared to those in the OLS model. In addition, coefficients for age bands 41–65 and 66 or over were statistically significant at the 5 % level and decreased with age.

Table 3 reports the relationship of self-reported long-standing health conditions and socio-demographic characteristics with the EQ-5D scores using the two-part regression model.

**Table 3:** Two-part model: the probability of reporting full health and the EQ-5D scores under full health

Explanatory variables	Model 3: two part Health conditions without socio-demographics		Model 3: two part Health conditions with socio-demographics	
	*β*	SE	*β*	SE
*Logistic regression: the likelihood of respondents reporting full health*				
Pain	−4.047***	0.166	−4.007***	0.166
Insomnia	−1.052***	0.149	−1.020***	0.150
Anxiety/nerves	−2.267***	0.147	−2.267***	0.148
Depression	−2.580***	0.178	−2.612***	0.179
Diabetes	−0.737***	0.115	−0.680***	0.116
Breathing problems	−0.569***	0.081	−0.520***	0.082
High blood pressure	−0.459***	0.067	−0.349***	0.068
Heart disease	−1.036***	0.124	−0.924**	0.125
Osteoarthritis	−2.551***	0.151	−2.474***	0.152
Stroke	−0.477**	0.214	−0.356*	0.216
Cancer	−0.730***	0.144	−0.664***	0.146
Number of comorbidities	0.208*	0.119	0.182	0.120
Control variables				
Age band 41–65			−0.181***	0.067
Age band 66 or over			−0.265***	0.077
Male			−0.072	0.090
Male* 41–65			0.136	0.108
Male* 66 or over			0.052	0.119
White			0.073	0.110
School qualifications			0.145***	0.051
Degree			0.322***	0.047
White collar			0.067	0.048
Currently employed			0.291***	0.044
Observations	13,955		13,955	
*p* value	0.000		0.000	
Likelihood ratio *χ* ^2^	5,597		5,767	
Pseudo *R* ^2^	0.290		0.299	
AIC	13,733		13,583	
BIC	13,832		13,756	
*Truncated OLS regression: EQ*-*5D scores of respondents reporting non full health*				
Pain	−0.234***	0.009	−0.226***	0.009
Insomnia	−0.071***	0.012	−0.070***	0.011
Anxiety/nerves	−0.098***	0.011	−0.096***	0.011
Depression	−0.185***	0.011	−0.186***	0.011
Diabetes	−0.069***	0.013	−0.065***	0.013
Breathing problems	−0.092***	0.010	−0.084***	0.010
High blood pressure	−0.041***	0.010	−0.032***	0.009
Heart disease	−0.052***	0.013	−0.045***	0.013
Osteoarthritis	−0.100***	0.010	−0.094***	0.010
Stroke	−0.071***	0.021	−0.064***	0.020
Cancer	−0.029	0.019	−0.022	0.018
Number of comorbidities	0.013	0.013	0.018	0.012
Control variables				
Age band 41–65			0.006	0.012
Age band 66 or over			0.016	0.014
Male			0.012	0.017
Male* 41–65			−0.007	0.020
Male* 66 or over			−0.007	0.022
White			0.002	0.019
School qualifications			0.028***	0.008
Degree			0.022**	0.009
White collar			0.044***	0.008
Currently employed			0.045***	0.008
Observations	7,359		7,359	
*p* value	0.000		0.000	
Wald *χ* ^2^	2,004		2,114	
Sigma	0.245		0.242	
AIC	−5,786		−5,888	
BIC	−5,689		−5,722	

Reference categories: no health condition, no comorbidity, age band 15–40, female, non-white, no qualification, below degree, blue collar, currently unemployed

*SE* standard error

* *p* < 0.1; ** *p* < 0.05; *** *p* < 0.01

In the first part of the model, the inclusion of socio-demographic characteristics had a significant impact on the comorbidity variable with it no longer being significant. The other coefficients were not significantly altered, but we focussed on the model with socio-demographics. Respondents with all long-standing health conditions were less likely to report full health, and all health conditions were statistically significant. Respondents with pain were least likely to report full health, followed by respondents with depression, osteoarthritis and anxiety/nerves. Respondents with pain were about four times less likely to report full health, followed by respondents with depression over two and half times, with osteoarthritis about two and half times and with anxiety/nerves about twice less likely to report full health compared to respondents with no health condition. In contrast, respondents with high blood pressure were about 35 % less likely to report full health compared to respondents with no health condition.

In relation to socio-demographic characteristics, the likelihood of respondents reporting full health decreased by age; the older the respondents, the less likely they were to report full health. Respondents with higher educational attainments were more likely to report full health compared to respondents with no qualifications. Currently employed respondents were also more likely to report full health compared to currently unemployed ones.

In the second part, inclusion of socio-demographic characteristics did not have a significant impact on the predicted EQ-5D scores. All self-reported long-standing health conditions were associated with lower predicted EQ-5D scores apart from cancer and all health conditions apart from cancer were statistically significant. The pattern in the magnitude of scores for these health conditions was slightly different from those in the OLS and Tobit models when focussing on the model with socio-demographics. Respondents with pain tended to have the lowest scores, followed by respondents with depression, anxiety/nerves and osteoarthritis. Furthermore, the magnitude of the negative impact of all health conditions was smaller than that in the OLS and Tobit models. Respondents with pain had 0.226 lower in scores, followed by respondents with depression 0.186 lower, with anxiety/nerves 0.096 lower and with osteoarthritis 0.094 lower in scores compared to those with no health condition. On the other hand, respondents with high blood pressure tended to have the highest scores, which reduced by 0.032 compared to those with no health condition. Number of comorbidities is not statistically significant.

In terms of socio-demographic characteristics, the pattern in the magnitude of scores remained similar to those in the OLS model. Respondents with higher educational attainments tended to have higher scores compared to respondents with no qualifications. White collar workers and currently employed respondents also tended to have higher scores than blue collar workers and currently unemployed ones respectively.

The fits from the two-part model cannot be directly compared to the fits of the OLS and Tobit models. However, a comparison can be made between the RMSE and sigma values produced by the three models. The comparison suggests that the OLS models produced the best performance, and the Tobit and two-part models produced similar, but worse performance.

## Discussion

We undertook this study to explore the relative impact of 11 long-standing health conditions on HRQoL in the UK population aged 15–86. We utilised one generic preference-based HRQoL measure—the EQ-5D. Overall, all models showed that pain, depression, osteoarthritis and anxiety/nerves had the largest negative impact on HRQoL. There were some differences in results using different models. In the OLS and Tobit models, respondents with pain reported the lowest HRQoL scores, followed by depression, osteoarthritis and anxiety/nerves. In the two-part model, anxiety/nerves had slightly larger negative impact on HRQoL than osteoarthritis. We also found that respondents with high blood pressure reported the highest HRQoL scores in all three models. These results remained consistent controlling for socio-demographic characteristics and other health conditions. Number of comorbidities was only statistically significant in the Tobit models, indicating that respondents with one or more comorbidities reported lower HRQoL scores, but the impact was smaller than for any long-standing health condition.

In relation to socio-demographic characteristics, all models reported that respondents with higher educational attainments, white collar workers (i.e. those with higher socio-economic status) and currently employed respondents tended to have higher HRQoL scores. The Tobit model also reported that retired respondents tended to report lower HRQoL scores.

The impact of long-standing health conditions on HRQoL has been increasingly acknowledged in terms of using utility-based HRQoL measures such as the EQ-5D, 15D, SF-6D, health utilities index, quality of well-being index and assessment of quality of life [11–13, 25–31]. Overall, these studies found that it was common to observe one or more comorbidities in their population surveys, and people with one or more comorbidities and lower socio-economic status and older people tended to have lower HRQoL scores. Our results were similar to the existing literature using the Tobit model. In addition, there have been numerous other HRQoL studies done using different methodologies. These studies were usually based on a single health condition, so were not able to control for the confounding effect of having other health conditions and socio-demographic characteristics. A person with diabetes, for example, is more likely to have other health conditions. Furthermore, these studies provided different estimates for a single long-standing health condition and were produced using different populations, different HRQoL measurement instruments and statistical analysis, so making comparisons was fraught with problems for policy making [32–38]. Comparisons with these previous studies may be difficult because of these differences in study populations. Our study represents an important contribution to the literature on the relative impact of different health conditions to health state utility values in the UK using a consistent instrument and after controlling for the confounding impact of other health conditions. They provide useful evidence for use in economic evaluations when assessing health care interventions in the UK.

On the other hand, we can compare our HRQoL scores to the two previous studies done on the UK population in relation to some socio-demographic characteristics. Both previous studies found that the oldest age group tended to report worse scores than younger age groups [39, 40]. Age variables were not significant in our OLS model though age variables had small significant coefficients in the Tobit model—which were consistent with previous literature. The small size of an age effect is supported by the analysis of the 2000 and 2002 MEPS by Sullivan and Ghushchyan [41] and this will account for why it was not significant in some models. Sullivan and Ghushchyan found, after controlling for health conditions and other socio-demographic characteristics, there was a significant age effect but it had a very small coefficient at 0.0003. Given that SYC (*N* = 13,955) had a much smaller sample size than MEPS (*N* = 37,933), then age variables were less likely to be significant. One of the previous studies also reported that people educated to degree level reported higher scores than those without a degree [40]. Our OLS and Tobit models produced similar results as the existing literature; however, we found that people educated to school level reported higher scores than those educated to degree level in our two-part model. In addition, the existing literature showed that women usually report a lower HRQoL than men [11, 12, 29]. However, there were no gender differences in our results after controlling for other variables.

The use of self-reported health status measures for clinical and policy purposes could be potentially increased by the available information on HRQoL from a large and nationally representative sample of the UK population, although the SYC data set was based on a response rate of only 17.8 %. The low response rate may have resulted in selection bias, but we do not know the direction or degree of the bias. A comparison with UK norms suggests that the sample is representative of the general population, but we cannot rule out the possibility that for some conditions those with, say, worse health were less likely to respond. We may treat these estimates as norms for the UK population and there is a highlighted need to take the impact of long-standing health conditions into account in both clinical and policy settings in light of the differences in the estimates of these health conditions. Almost all of long-standing health conditions examined were statistically significant, particularly for pain, depression, osteoarthritis and anxiety/nerves.

There are a number of limitations in our study. First, the SYC data set was cross-sectional; hence, we cannot derive causal relationships between long-standing health conditions and HRQoL; instead, we can only interpret our results as an association between them. Second, the SYC data set only had self-reported, long-standing health conditions, where respondents were given prompting from a limited number of health conditions included in the questionnaire. By relying on these self-reported health conditions, biased estimates of the prevalence of these conditions may have occurred in our study, and Wu et al. [42] found that this was the case particularly for less educated people. Third, we used the SYC data set with deletion of incomplete responses, which had slightly healthier respondents (with a mean EQ-5D score of 0.831) than the whole sample of the intended respondents (0.817). Fourth, when measuring the health status of the UK general population, a ceiling effect may have occurred since there are only three response categories for each of the five questions in the utility-based HRQoL measure (i.e. the EQ-5D) [25, 43]. That is, the EQ-5D may not be fully able to distinguish between respondents whose health statuses are at the upper end of the scale.

In relation to the regression models we used, the OLS model produced the best performance, but it ignored the bounded nature of the EQ-5D scores (−0.594 to 1) and could have resulted in biased estimates. The Tobit model produced correct estimates of the effects on the mean only if the error terms were in normal distribution with the uniform variance [44]. There was some flexibility and computational simplicity in the two-part model because of providing two independent parts—the probability of respondents reporting full health and the predicted EQ-5D scores of respondents reporting non-full health. This also gives additional information by looking at the two parts separately. For example, from the second part, cancer is not statistically significant, we can see that this is driven by the reported decrement for respondents not in full health. However, this provision caused a constraint on the second part of the model. That is, respondents with non-full health were not randomly selected from the whole sample controlling for regressors; therefore, selection bias may have occurred in the second-part regression results.

In conclusion, our results showed that among the 11 long-standing health conditions, pain, depression, osteoarthritis and anxiety/nerves led to the greatest losses of HRQoL in the UK population. Furthermore, there was a statistical decrease in HRQoL with one or more comorbidities. Potential policy implication is that our findings should be taken into account in economic evaluations when assessing health care interventions. However, whilst this is evident in the cross-sectional data set, additional research using longitudinal data sets is required to observe whether this evidence remains over time and if so a causality relationship between long-standing health conditions and HRQoL could be established. Further research is also needed using clinically diagnosed long-standing health conditions.

## Appendix

See Table 4.

**Table 4:** Variables used in analyses

Variables	Definitions
*Dependent variable*	
EQ-5D score	A general quality of life measure; scores range from −0.594 to 1. (i.e. worse than being dead—full health)
*Independent variables*	
Long-standing health conditions	
Pain	=1 if yes
Insomnia	=1 if yes
Anxiety/nerves	=1 if yes
Depression	=1 if yes
Diabetes	=1 if yes
Breathing problems	=1 if yes
High blood pressure	=1 if yes
Heart disease	=1 if yes
Osteoarthritis	=1 if yes
Stroke	=1 if yes
Cancer	=1 if yes = *0 reference category: no*
Number of comorbidities	=1 if yes = 0 *reference category: no*
Socio-demographic characteristics	
Age bands	
Younger (<41)	=1 if aged <41 *reference category*
Middle aged (41–65)	=1 if aged 41–65
Retired (66 or over)	=1 if aged 66 or over
Male	=1 if male *reference category: female*
Interaction terms	
Male*Age band <41)	=1 if male aged <41 *reference category*
Male*Age band (41–65)	=1 if male aged 41–65
Male*Age band (66 or over)	=1 if male aged 66 or over
White	=1 if white *reference category: non*-*white*
Educational attainment	
School qualifications	=1 if yes *reference category: no qualifications*
Degree	=1 if yes *reference category: below degree*
Socio-economic status	
White collar	=1 if yes *reference category: blue collar*
Current employment status	=1 if yes
